# Transcriptomic Correlates of Immunologic Activation in Head and Neck and Cervical Cancer

**DOI:** 10.3389/fonc.2021.714550

**Published:** 2021-10-06

**Authors:** Cristina Saiz-Ladera, Mariona Baliu-Piqué, Francisco J. Cimas, Aránzazu Manzano, Vanesa García-Barberán, Santiago Cabezas Camarero, Gonzalo Fernández Hinojal, Atanasio Pandiella, Balázs Győrffy, David Stewart, Juan J. Cruz-Hernández, Pedro Pérez-Segura, Alberto Ocana

**Affiliations:** ^1^ Experimental Therapeutics Unit, Medical Oncology Department, Hospital Clínico Universitario San Carlos (HCSC), Instituto de Investigación Sanitaria (IdISSC), Madrid, Spain; ^2^ Translational Oncology Laboratory, Centro Regional de Investigaciones Biomedicas, Castilla‐La Mancha University (CRIB‐UCLM), Albacete, Spain; ^3^ Instituto de Biología Molecular y Celular del Cáncer and Centro de Investigación Biomédica en Red de Cáncer (CIBERONC), Centro Superior de Investigaciones Científicas (CSIC), Salamanca, Spain; ^4^ Department of Bioinformatics, Faculty of Medicine, Semmelweis University, Budapest, Hungary; ^5^ 2nd Department of Pediatrics, Faculty of Medicine, Semmelweis University, Budapest, Hungary; ^6^ Institute of Enzymology, Research Centre of Nature Sciences, Budapest, Hungary; ^7^ Ottawa University Hospital, University of Ottawa, Ottawa, ON, Canada

**Keywords:** head and neck squamous cell carcinoma (HNSCC), human papillomavirus, transcriptome signature, immune gene signatures, cervical squamous cell carcinoma (CSCC)

## Abstract

Targeting the immune system has emerged as an effective therapeutic strategy for the treatment of various tumor types, including Head and Neck Squamous Cell Carcinoma (HNSCC) and Non-small-Cell Lung Cancer (NSCLC), and checkpoint inhibitors have shown to improve patient survival in these tumor types. Unfortunately, not all cancers respond to these agents, making it necessary to identify responsive tumors. Several biomarkers of response have been described and clinically tested. As of yet what seems to be clear is that a pre-activation state of the immune system is necessary for these agents to be efficient. In this study, using established transcriptomic signatures, we identified a group of gene combination associated with favorable outcome in HNSCC linked to a higher presence of immune effector cells. *CD2*, *CD3D*, *CD3E*, and *CXCR6* combined gene expression is associated with improved outcome of HNSCC patients and an increase of infiltrating immune effector cells. This new signature also identifies a subset of cervical squamous cell carcinoma (CSCC) patients with favorable prognosis, who show an increased presence of immune effector cells in the tumor, which outcome shows similarities with the HP-positive HNSCC cohort of patients. In addition, *CD2*, *CD3D*, *CD3E*, and *CXCR6* signature is able to predict the best favorable prognosis in terms of overall survival of CSSC patients. Of note, these findings were not reproduced in other squamous cell carcinomas like esophageal SCC or lung SCC. Prospective confirmatory studies should be employed to validate these findings.

## Introduction

Squamous Cell Carcinoma (SCC) includes a wide range of tumors originated from diverse anatomical locations that share common molecular and genetic features (1). SCCs arise from squamous and non-squamous epithelial tissues, and they are classified according to their location as head and neck, esophagus, lung, and cervix, among others (2). SCCs are in many occasions incurable diseases particularly in their advanced stages (1). This is the case for Head and Neck Squamous Cell Carcinoma (HNSCC) and Cervical Squamous Cell Carcinoma (CSCC), since the therapeutic options for both tumors, when diagnosed in the metastatic setting, are limited and outcome is severely compromised (3, 4). In both tumors, human papilloma virus (HPV) infection plays a central oncogenic role in a substantial proportion of cases and associates with aggressiveness and clinical outcome particularly in HNSCC (5). The classical treatment for HNSCC includes chemotherapies based on platinum agents and taxanes combined with anti-EGFR antibodies, which has demonstrated to improve survival (3). Recently, immunomodulators, particularly immune checkpoint inhibitors like pembrolizumab or nivolumab, have shown to improve relapse-free survival (RFS) and overall survival (OS) (5–8). Although this has dramatically changed the expected survival of HNSCC patients, the metastatic setting, nevertheless, remained an incurable condition (9). In a similar manner, immunotherapy has shown efficacy in CSCC patients with recurrent or metastatic cancers with disease progression or after chemotherapy when tumors express PD-L1 (Combined Positive Score, CPS ≥1) (10). In other tumor types like non-small-cell lung (NSCLC) or bladder cancer, checkpoint inhibitors have also demonstrated to provide clinical efficacy (6–8, 11). However, blocking of immune inhibitory signals with antibodies against PD1 or PD-L1 does not always result in clinical response (12, 13). Activation of the immune system, including the presence of effector T cells in the tumor, is a main requisite for these therapies to be effective (14). In addition, expression of PD1 or PD-L1 or the presence of Tumor Infiltrating Lymphocytes (TIL) is associated with favorable survival, independently of the therapy administered, confirming the relevant role of the immune system in the antitumoral action (15, 16).

Identification of genomic correlations of immune activation is an approach that could permit the selection of tumors susceptible to respond to immunotherapies. In this context, the mutational burden or altered mismatch repair mechanisms have been described as predictors of response to immunotherapies in several types of tumors (17–19). Likewise, some molecular alterations have been described as linked to the lack of activity of immunotherapeutic agents including JAK2 or B2M mutations (20). Regardless, recognition of immune pre-activated tumors, usually associated with favorable prognosis, is a requisite for most immune therapies to be efficient.

In this article we explored gene sets that predict favorable prognosis in HNSCC, with the aim to identify pre-activated immune tumors. We identified a transcriptomic signature associated with favorable outcome and linked with the infiltration of effector immune cells in this tumor. Similar findings were observed in Cervical SCC (CSCC), confirming its relevance.

## Material and Methods

### Immune Gene Signatures

Previously described immune signatures, i.e., expanded immune gene signature (CD3D, IL2RG, CXCL10, IDO1, NKG7, CIITA, HLA-E, HLA-DRA, CD3E, CXCR6, STAT1, CCL5, LAG3, GZMK, TAGAP, CD2, STAT1, CXCL13, GZMB), IFN gamma signature (IDO1, CXCL10, CXCL9, HLA-DRA, IRF9, IFNG, STAT1), cytotoxic T lymphocyte (CTL) signature (CD8A, CD8B, GZMA, GZMB, and PRF1), and HLA genes (HLA-A, HLA-B) were used to study the prognostic capacity of the genes composing each signature (21–23).

### Clinical Outcome Analysis of Individual Genes and Signatures

The KM Plotter Online Tool (http://www.kmplot.com) (24, 25) was used to explore the relationship between the expression of described gene signatures (expanded immune gene signature, IFN gamma signature, CTL signature, and HLA genes) and the newly identified signature (*CD2*, *CD3D*, *CD3E*, and *CXCR6*) with patient clinical outcomes. We evaluated the prognostic values of mRNA expression of previously described gene signatures, for overall survival (OS) in a cohort of HNSCC patients (n=527) in all stages from the Cancer Genome Atlas (TCGA) database.

Briefly, publicly available RNA-seq HTSeq count files obtained from Illumina HiSeq 2000 RNA Sequencing Version 2 platform were analyzed for quantification of mRNA expression. Negative binomial distribution method was used through DESeq package to normalize the raw count data, and Bioconductor AnnotationDbi package (http://bioconductor.org/packages/AnnotationDbi/) was employed to annotate Ensembl transcript IDs with gene symbols (n = 25,228). After that, second scaling normalization was performed to calculate the mean expression of all genes in each patient sample to 1,000 to reduce batch effects.

In order to determine the correlation between gene expression and OS, Cox proportional hazards regression analysis was performed by using the Survival R package v2.38 (http://CRAN.R-project.org/package=survival/). Log-rank P values, hazard ratios (HR), and 95% confidence intervals (CI) were calculated. In terms of statistical analysis, false discovery rate (FDR) was computed to correct for multiple hypothesis testing, and the result was only accepted as significant in the case of FDR < 10%. Each possible cutoff was evaluated between the highest and lowest quartile of expression, and the best performing threshold with the lowest p value was used in the final analysis when drawing the Kaplan–Meier plot. In addition, multivariate survival analysis was performed for the gene expression and clinical features to assess independence from known epidemiological and clinical variables, including race, sex, age, tumor stage, and tumor grade when available. Finally, only genes associated with good outcome (HR<0.65, p<0.05, and FDR≤ 5%) were selected after screening through KM Plotter.

According to the results, the gene expression of the individual genes and the newly identified signature (*CD2*, *CD3D*, *CD3E*, and *CXCR6*) were assessed for OS in the different cohorts including HNSCC (n= 527), Esophageal SCC (n=81), Lung SCC (n=501), and Cervical SCC (n=254). In case we identified an association with multiple genes, the mean expression of the selected genes was used. Patients were divided according to the best cutoff values of the gene expression [lowest p-value (*p*)] into high *vs* low expression.

For graphical representation, a heatmap plot was performed using *GraphPad Prism 8.0* tool. Survival HR parameter was represented as labels overlaid on the graph. The scale color meaning was represented as follows: blue, favorable outcome; red, detrimental outcome. Detailed information about the patients and clinical variables that were included in this study are resumed in **Table 1**.

**Table 1:** Patients’ clinical characteristics.

Clinical data available	HNSCC		Lung SCC		CSCC	
***Total n***	527		504		254	
***Median age***	61 years		NA		47 years	
***Gender (male)***	73%		74%		0%	
***Smoking history***	76%		96%		NA	
***Alcohol history***	68%		NA		NA	
***Median follow-up***	21.4 months		22.2 months		22.4 months	
***Stage***	***n***	***%***	***n***	***%***	***n***	***%***
***Stage 1***	27	5.90%	246	48.90%	126	51.10%
***Stage 2***	74	16.30%	165	32.80%	62	25.10%
***Stage 3***	82	18.10%	85	16.90%	43	17.40%
***Stage 4***	270	59.60%	7	1.40%	16	6.50%
***Grade***	***n***	***%***	***n***	***%***	***n***	***%***
***Grade 1***	63	12.50%	NA	NA	12	5.30%
***Grade 2***	311	61.50%	NA	NA	110	48.70%
***Grade 3***	125	24.70%	NA	NA	103	45.60%
***Grade 4***	7	1.38%	NA	NA	1	0.40%
***Stage***	***n***	***%***	***n***	***%***	***n***	***%***
***T1***	NA	NA	114	22.62%	111	56.35%
***T2***	NA	NA	295	58.53%	58	29.44%
***T3***	NA	NA	71	14.09%	19	9.64%
***T4***	NA	NA	24	4.76%	9	4.57%
***Lymph Node***	***n***	***%***	***n***	***%***	***n***	***%***
***N0***	NA	NA	320	64.26%	105	67.31%
***N1***	NA	NA	133	26.71%	51	32.69%
***N2***	NA	NA	40	8.03%	0	0%
***N3***	NA	NA	5	1.00%	0	0%
***Metastasis***	***n***	***%***	***n***	***%***	***n***	***%***
***M0***	NA	NA	414	98.34%	102	93.58%
***M1***	NA	NA	7	1.66%	7	6.42%

Epidemiological data including age (median years), gender, smoking and alcohol history (in corresponding percentages, %), and median follow-up (in months) of included patients per each SCC type in this study are displayed in the table. In addition, the corresponding total number (n) and the percentage (%) of included patients of each SCC type are showed by stage (1 to 4), grade (1 to 4), and TNM staging system (T1 to T4, N0 to N3, and M0 to M1).

NA, Not Available.

### Analysis of Tumor Mutational Burden and HPV

Clinicopathological characteristics of patients, including stage, grade, sex, race, including tumor mutational burden (TMB), were available and allowed to restrict the analysis in the cited KM Plotter Online Tool (http://www.kmplot.com).

The TMB was determined from whole-exome sequencing data from TCGA datasets used as the number of genes with a mutation. A gene was assigned “mutated” in case it had, at least, one mutation. Then, the median number of mutations across all samples within each tumor type was determined and was used as a cutoff: samples that had more “mutated” genes were determined as “high TMB,” and samples that had fewer “mutated” genes were assigned to the “low TMB” cohort.

In terms of HPV status detection, the Cancer Genomic Atlas (TCGA) dataset used the HPV16 DNA genotyping and mRNA expression to detect HPV oncoprotein transcripts.

### Association Between Tumor Immune Infiltrates and Gene Expression

The correlation between gene expression and the presence of tumor immune infiltrates (CD8^+^T cells, NK cells, macrophages, and dendritic cells) in HNSCC and CSCC was analyzed using the Tumor Immune Estimation Resource (TIMER 2.0) platform (http://cistrome.org/TIMER/) (26, 27), a dataset that contains 10,897 samples from diverse cancer types available in the TCGA database. To analyze the relationship between tumor gene expression and immune infiltration, the available “Gene Module” from TIMER 2.0 was used. TIMER 2.0 uses an R package that integrates six computational algorithms to associate the tumor immune infiltrate populations with genomic and transcriptomic changes in the tumors (based on microarrays or RNAsequencing data), providing an estimation of immune infiltration levels for TCGA database or user-provided tumor profiles. To make the estimations of the immune cell populations, the cited algorithms are based on gene signature-based approaches utilizing a list of cell-type-specific gene sets and using the expression values of these signature gene sets in tissue samples. Specific tissue types, distinct cancer-cell intrinsic gene expression, and different immune cell types are considered to establish Spearman’s correlations between the expression of the input gene and the abundance of the immune cell type as well as its subtypes across cancer types under study. These algorithms were applied to the expression profiles of the Cancer Genome Atlas (TCGA) tumors, allowing to explore various associations between immune infiltrates and genetic features in the TCGA cohorts. The association between the immune infiltrates and the clinical features, such as HPV infection condition, was possible sorting patient cohorts in case of HNSCC. The results are displayed as a functional heatmap, and by clicking on each box of the heatmap, subsequently it generates a scatter plot showing the association of the gene expression with the infiltrated immune cell type. “Purity adjusted” option was selected (the correlation of the given gene expression with tumor purity as proportion of cancer cells in a sample), and the most immune cell types are negatively correlated with tumor purity (data not shown). Partial Spearman’s correlation was used to perform this association analysis, and statistical significance was expressed (*p*<0.05). Correlation value was displayed by “Rho” parameter. Positive correlation is associated with Rho>0 values, and negative correlation is associated with Rho<0 values.

## Results

### Evaluation of Immune Activated Signatures and Clinical Outcome in HNSCC

**Figure 1:**
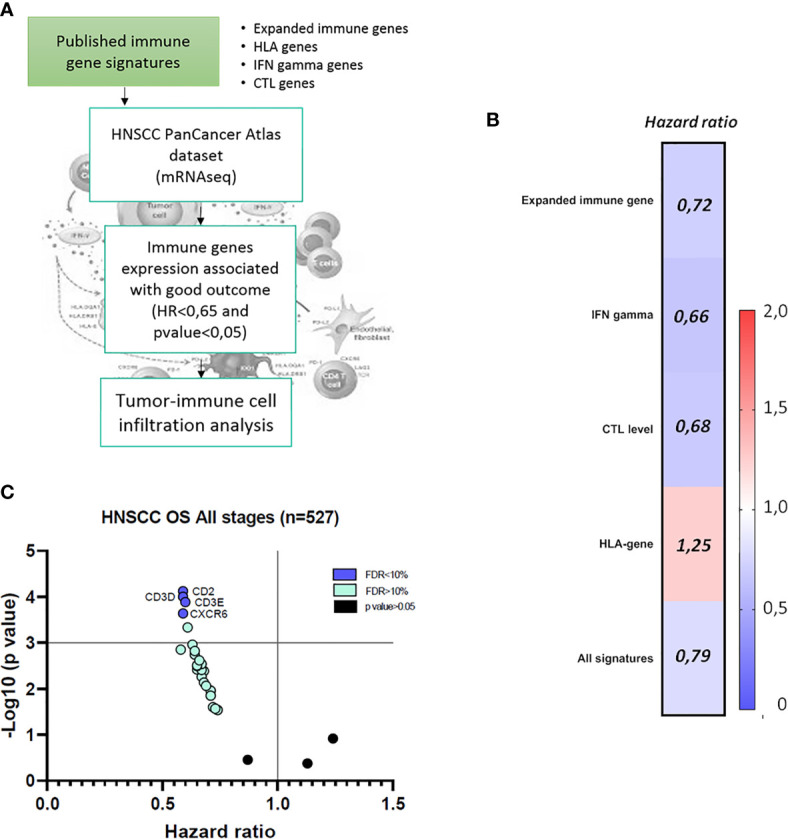
Evaluation of the impact of immune gene signatures (*Expanded* immune genes, *IFN gamma genes*, *CTL level*, and *HLA genes*) on clinical outcome in Head and Neck Squamous Cell Carcinoma (HNSCC) patients. **(A)** Flow chart of the analysis of the four published immune gene signatures and the transcriptomic expression effect in HNSCC patients’ clinical outcome and the correlation with tumor immune cell infiltrate composition. **(B)** Hazard ratio (HR) heat map for risk of death of each published immune gene signatures (*Expanded immune genes*, *IFN gamma genes*, *CTL levels*, and *HLA genes)* and combined, in association with overall survival (OS) in HNSCC patients at all stages (n = 527), using data from TCGA database as described in *Materials and Methods*. HR < 1 discriminates a risk reduction. Blue color represents favorable prognosis, and red color represents detrimental prognosis, with 95% confidence interval (CI) and *p* value < 0.05. **(C)** Graph representation of immune genes with the most favorable outcome in HNSCC (HR < 1, *p* value < 0.05 and FDR < 10%), in blue spots. Green spots represent immune genes with good prognosis and FDR > 10% and p < 0.05. Black spots represent immune genes without statistical significance (p value > 0.05).

With the main goal to identify immunologic correlates associated with prognosis in HNSCC, we took advantage of previous published immune transcriptomic signatures (21–23). We first explored the association of the genes included in each signature with favorable prognosis, and latter their correlation with immune populations as described in *Materials and Methods* (**Figure 1A**). The correlation analysis between each immune signature and the 32 individual genes with OS is displayed as a heatmap in **Figure 1B** and **Table 2**. Favorable survival was observed for the expanded immune genes signature (HR=0.72; 95% confidence intervals CI=0.55–0.94; log rank p=0.016), the IFN gamma signature (HR=0.66; 95% CI=0.50–0.87; log rank p=0.0028), and the CTL level signature (HR=0.68; 95% CI=0.52–0.89; log rank p=0.0053) (**Figure 1B**). Only the HLA signature predicted unfavorable survival (HR=1.25; 95% CI=0.92–1.70; log rank p=0.150) (**Figure 1B**). For the whole population of tumors (all stages, n=527), most individual genes were associated with favorable outcome (**Table 2**). The different gene signatures did not predict for better OS in comparison with some individual transcripts, particularly with CD2 (HR=0.59; 95% CI=0.45–0.77; log rank p= 7.5e−05), CD3D (HR=0.59; 95% CI=0.45–0.77; log rank p=0.0001), CD3E (HR=0.60; 95% CI=0.46–0.78; log rank p=0.00013), and CXCR6 (HR=0.59; 95% CI=0.45–0.78; log rank p=0.00023) (**Table 2**). In addition, these individual genes presented a smaller FDR (FDR<10%) compared with the other analyzed genes and signatures (**Figure 1C**).

**Table 2:** Clinical outcome of individual immune genes from the four immune gene signatures in terms of overall survival (OS) of HNSCC patients.

Signature	OS N = 527 (All stages)				
	Gene symbol	HR	CI	pValue	FDR
***Expanded*** ***immune*** ***genes***	CXCL13	0,58	0,41–0,81	0,0014	50%
	**CD2**	**0,59**	**0,45–0,77**	**0,000075**	**5%**
	**CD3D**	**0,59**	**0,45–0,77**	**0,0001**	**5%**
	**CXCR6**	**0,59**	**0,45–0,78**	**0,00023**	**2%**
	**CD3E**	**0,60**	**0,46–0,78**	**0,00013**	**5%**
	GZMK	0,61	0,46–0,81	0,00046	20%
	GZMB	0,63	0,48–0,83	0,0011	20%
	IL2RG	0,64	0,48–0,85	0,0018	10%
	TAGAP	0,65	0,49–0,87	0,0032	50%
	LAG3	0,67	0,51–0,89	0,0055	50%
	NKG7	0,67	0,52–0,88	0,0039	50%
	CIITA	0,67	0,51–0,87	0,0029	50%
	CCL5	0,68	0,52–0,9	0,0073	>50%
	IDO1	0,71	0,54–0,92	0,011	>50%
	CXCL10	0,71	0,54–0,93	0,014	>50%
	HLA-DRA	0,73	0,55–0,97	0,027	>50%
	STAT1	0,74	0,56–0,97	0,029	>50%
	HLA-E	0,87	0,65–1,16	0,35	100%
***IFN gamma*** ***genes***	CXCL9	0,65	0,49–0,87	0,0038	>50%
	IFNG	0,68	0,52–0,88	0,0041	>50%
	IDO1	0,71	0,54–0,92	0,011	>50%
	CXCL10	0,71	0,54–0,93	0,014	>50%
	IRF9	0,72	0,53–0,96	0,025	>50%
	HLA-DRA	0,73	0,55–0,97	0,027	>50%
	STAT1	0,74	0,56–0,97	0,029	>50%
***CTL level***	GZMB	0,63	0,48–0,83	0,0011	20%
	GZMA	0,64	0,49–0,85	0,0015	50%
	PRF1	0,65	0,48–0,86	0,0031	>50%
	CD8A	0,66	0,5–0,86	0,0024	50%
	CD8B	0,69	0,52–0,91	0,0086	>50%
***HLA genes***	HLA-B	1,13	0,84–1,5	0,42	100%
	HLA-A	1,24	0,95–1,61	0,12	100%

Table shows the list of individual genes belonging to the four previous published immune gene signatures. HR < 1 discriminates a risk reduction. The 95% confidence interval (CI) and p value are displayed. p value < 0.05 and FDR (False Discovery Rate) to correct for multiple hypothesis testing are displayed.

The bold values represent those genes that stand out with the best clinical outcome (HR < 0.65, FDR < 10%, p < 0.05).

Considering these results, we decided to analyze the gene set combination of *CD2*, *CD3D*, *CD3E*, and *CXRC6*.

The expression of PDL1 or PD1 is associated with favorable clinical outcome and improved response to immunotherapy including HNSCC tumors (5–8). We identified a positive correlation between the new immune gene signature (*CD2*, *CD3D*, *CD3E*, *CXCR6*) and *CD274* (*PD-L1*) expression in HNSCC patients (Spearman rank correlation coefficient = 0.56; P=2.4E^-43^), which could support the outcome prediction in this group.

### The Combined Gene Signature *CD2*, *CD3D*, *CD3E*, and *CXCR6* Predicted Favorable Prognosis in Different HNSCC Clinical Stages

**Figure 2:**
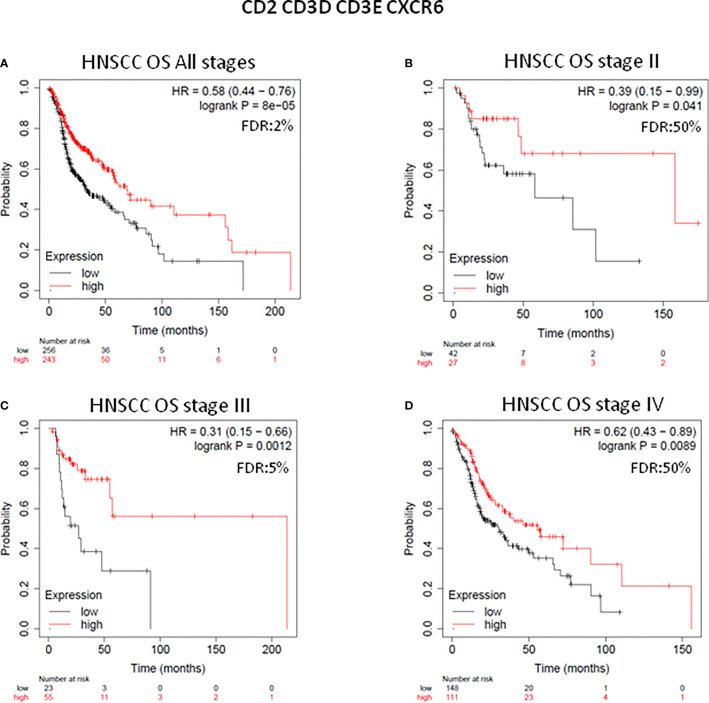
Clinical outcome analysis of *CD2*, *CD3D*, *CD3E*, *CXCR6* immune gene-set combination in each stage of HNSCC patients. Survival plots of the combination of four immune genes with the most favorable prognosis (HR < 0.65, *p* value < 0.05, and FDR < 10%) at all stages of HNSC patients (n = 527) **(A)**, at stage II (n = 69) **(B)**, at stage III (n = 78) **(C)**, and at stage IV (n = 259) **(D)** are displayed. Patients whose tumors harbor high gene expression levels predicted better survival (red line), and those with low gene expression levels predicted worse survival (black line). Number of patients at risk at every time (months), with high (in red) and low gene expression (in black) are displayed. HR for risk of death and OS are displayed. HR < 0.65 discriminates a risk reduction. FDR is also displayed. The gene combination is displayed at the top of the figure.

Next, we tested whether a new signature composed by *CD2*, *CD3D*, *CD3E*, and *CXRC6* could improve the potential prediction capacity in HNSCC patients. The combined immune signature demonstrated a higher prediction in the stage II and III patient subgroups, even with a small number of patients: for stage II (n=69), HR=0.39; 95% CI=0.15–0.99; log rank p=0.041; and stage III subgroup: (n=78), HR=0.31; 95% CI=0.15–0.66; log rank p=0.0012). For all stages the combined signature also predicted favorable survival: (n=527), HR=0.58; 95% CI=0.44–0.76; log rank p=8e^-05^). Results in stage IV were also significant but with less magnitude of benefit compared with the other subgroups: (n=259), HR=0.62; 95% CI=0.43–0.89; log rank p=0.0089) (**Figure 2**).

### *CD2*, *CD3D*, *CD3E*, and *CXCR6* Expression Is Associated With Infiltration of Memory CD8+ T, Activated NK, Dendritic Cells, and M1 Macrophages in HPV-Positive HNSCC

We next explored the association of the expression at an individual transcriptomic level of *CD2*, *CD3D*, *CD3E*, and *CXRC6* with the presence of tumor-infiltrating immune cell populations. All genes had a negative correlation with tumor purity, demonstrating the high presence of immune populations.

**Figure 3:**
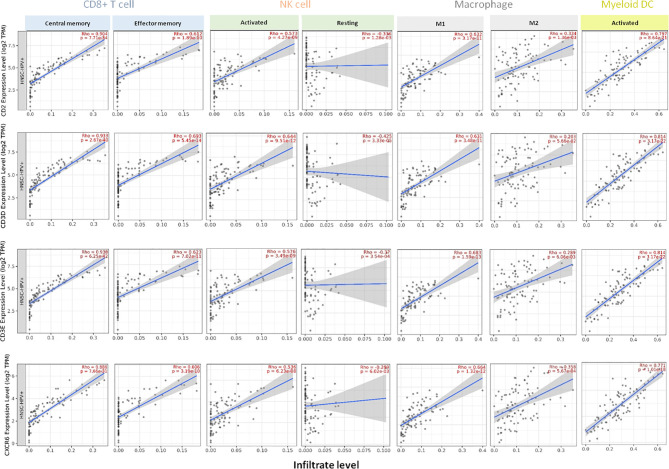
Association of the expression of *CD2*, *CD3D*, *CD3E*, *CXCR6* with specific tumor-infiltrating immune cell populations in HPV-positive HNSCC patients. Expression of each gene and association with the presence of CD8+ T cells (central memory and effector memory subsets), NK cells (activated and resting), macrophages (M1 and M2 types), and activated myeloid dendritic cells in HPV-positive HNSCC (n = 98). Each panel presents the relationship between infiltrates estimation value with the purity-adjusted spearman’s correlation parameter Rho and gene expression join p value < 0.001. Rho value > 0 represents a positive correlation, and Rho < 0 represents a negative correlation. Association with immune cell populations was provided by *TIMER 2.0* software and was correlated with transcriptome expression level of each immune gene, as described in *Material and Methods*.

In HPV-positive HNSCC tumors (n=98), we observed the strongest positive correlation with central memory CD8^+^ T cell subpopulation (*CD2:* Rho=0.904, *CD3D:* Rho=0.933, *CD3E:* Rho=0.938 and *CXCR6:* Rho=0.886) and also a positive correlation with effector memory CD8^+^ T cells (*CD2:* Rho=0.612, *CD3D:* Rho=0.693, *CD3E:* Rho=0.623 and *CXCR6:* Rho=0.606) (**Figure 3**). In the case of NK cells, the stronger association was found for activated NK cells (*CD2:* Rho=0.573, *CD3D:* Rho=0.644, *CD3E:* Rho=0.576 and *CXCR6:* Rho=0.536), compared with a negative association with resting NK cells (*CD2:* Rho=−0.336*, CD3D:* Rho=−0.425*, CD3E:* Rho=−0.370 and *CXCR6:* Rho=−0.289). Regarding the macrophage population, HPV-positive tumors showed a positive correlation with both subtypes (M1 and M2): M1 (*CD2:* Rho=0.632, *CD3D:* Rho=0.631, *CD3E:* Rho=0.683, and *CXCR6:* Rho=0.664), and less significant for the M2 subtype (*CD2:* Rho=0.334, *CD3D:* Rho=0.203, *CD3E:* Rho=0.289, and *CXCR6:* Rho=0.358) (**Figure 3**). For the activated dendritic cell population, a strong correlation was observed (*CD2:* Rho*=0.797*, *CD3D:* Rho=0.814, *CD3E:* Rho=0.814, and *CXCR6:* Rho=0.771) (**Figure 3**).

**Figure 4:**
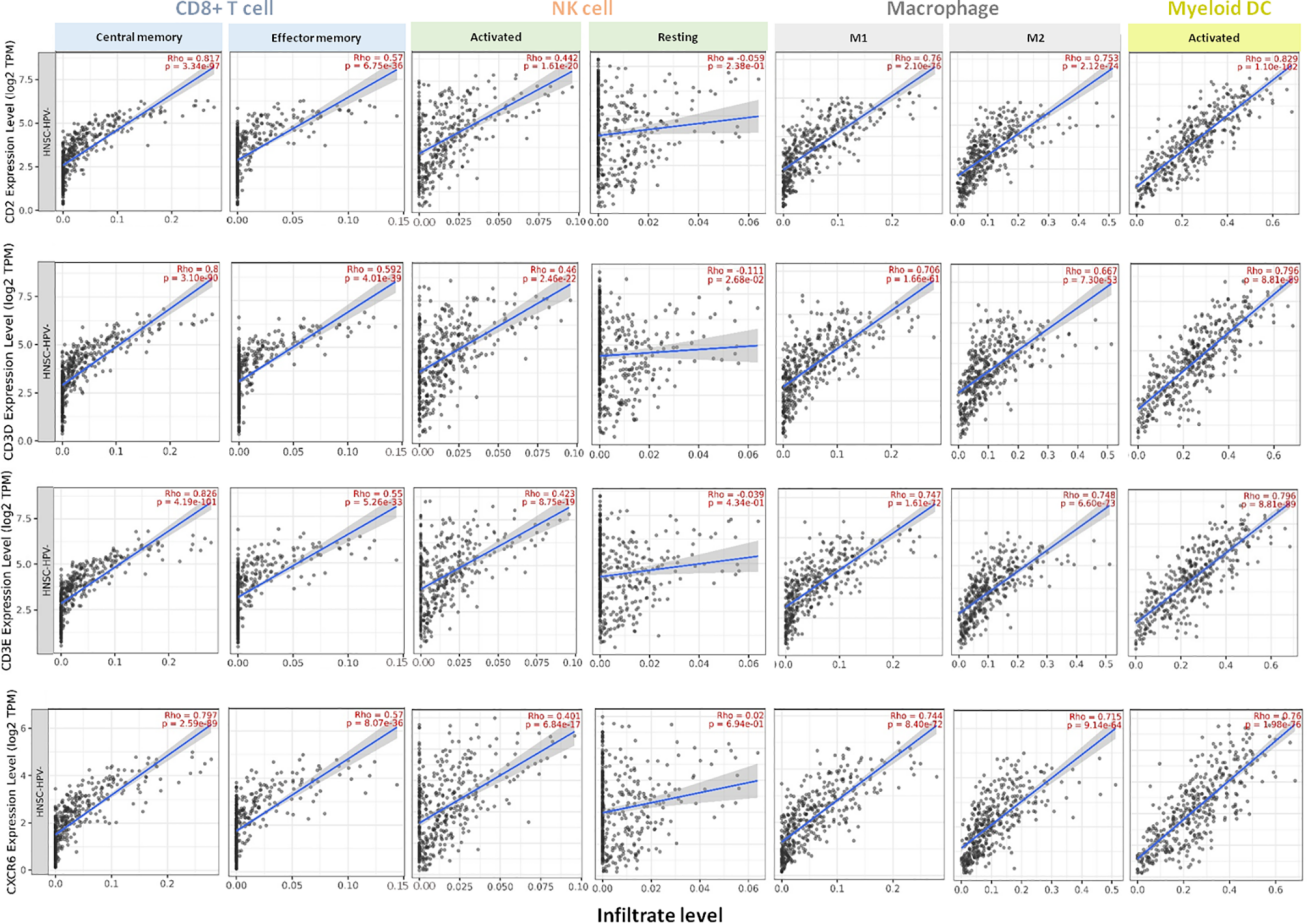
Association of the expression of *CD2*, *CD3D*, *CD3E*, *CXCR6* with specific tumor-infiltrating immune cell populations in HPV-negative HNSCC patients. Expression of each gene and association with the presence of CD8+ T cells (central memory and effector memory subsets), NK cells (activated and resting), macrophages (M1 and M2 types), and activated myeloid dendritic cells in HPV-negative HNSCC (n = 422). Each panel presents the relationship between infiltrates estimation value with the purity-adjusted spearman’s correlation parameter Rho and gene expression join p value < 0.001. Rho value > 0 represents a positive correlation, and Rho < 0 represents a negative correlation. Association with immune cell populations was provided by *TIMER 2.0* software and was correlated with transcriptome expression level of each immune gene.

In case of HPV-negative HNSCC tumors (n=422), we found a positive correlation between the gene expression level of *CD2*, *CD3D*, *CD3E*, and *CXRC6*, and CD8^+^ T cells subpopulations, finding the higher correlation with CD8+ central memory cells (*CD2:* Rho=0.817, *CD3D:* Rho=0.800, *CD3E:* Rho=0.826, and *CXCR6:* Rho=0.797). A less significant association was observed for the CD8+ effector memory cells (*CD2:* Rho=0.570, *CD3D:* Rho=0.592, *CD3E:* Rho=0.550, and *CXCR6:* Rho=0.570) (**Figure 4**). In addition, we found a positive association between the gene expression level of *CD2*, *CD3D*, *CD3E*, and *CXRC6* and the activated fraction of NK cells present in the tumor (*CD2:* Rho=0.442, *CD3D:* Rho=0.460, *CD3E:* Rho=0.423, and *CXCR6:* Rho=0.401). A negative association with resting NK cells (*CD2:* Rho=−0.059, *CD3D:* Rho=−0.111, *CD3E:* Rho=−0.039, and *CXCR6:* Rho=0.02) was observed. Similar findings were observed for M1 macrophage infiltrates (*CD2:* Rho=0.760, *CD3D:* Rho=0.706, *CD3E:* Rho=0.747, and *CXCR6:* Rho=0.744) and M2 macrophages (*CD2:* Rho=0.753, *CD3D:* Rho=0.667, *CD3E:* Rho=0.748, and *CXCR6:* Rho=0.715), being the association with M2 macrophages higher than those observed for HPV-positive HNSCC tumors. Finally, we analyzed the activation state of the myeloid dendritic cell subpopulation, observing a high correlation with activated dendritic cells (*CD2:* Rho=0.829, *CD3D:* Rho=0.796, *CD3E:* Rho=0.796, and *CXCR6:* Rho=0.760) (**Figure 4**).

### Evaluation of Immune Activated Genes in Other Squamous Cell Tumors

SCCs arise in a different locations and share molecular and genetic alterations (1). In this context, we decided to explore if the expression of four previous published immune gene signatures (Expanded immune genes, IFN gamma genes, CTL genes, and HLA) and the new identified *CD2*, *CD3D*, *CD3E*, and *CXCR6* gene combination were able to classify patients with different outcome in lung, esophageal, and cervical SCC. Unexpectedly, no significant effect of four previous published immune gene signatures was observed in lung SCC (data not shown), neither in the case of the evaluated 32 individual genes (**Table 3**) or in case of the new *CD2*, *CD3D*, *CD3E*, and *CXCR6* signature: (n=504), HR=0.85, 95% CI=0.64−1.13; log rank p=0.27 (**Supplementary Figure 1**). However, a detrimental outcome was observed for both, the 32 individual genes (**Table 4**), and the new signature in esophageal SCC (n=81; HR=2.92; 95% CI=1.19−7.18; log rank p=0.015) (**Supplementary Figure 1**).

**Table 3:** Clinical outcome of individual immune genes from the four immune gene signatures in terms of overall survival (OS) of LSCC patients.

Signature	OS N = 504 (All stages)				
	Gene symbol	HR	CI	pValue	FDR
***Expanded*** ***immune*** ***genes***	CXCL13	0,79	0,6–1,05	0,1	100%
	CD2	0,80	0,63–1,09	0,19	100%
	CD3D	0,83	0,6–1,1	0,2	100%
	CXCR6	0,84	0,62–1,14	0,27	100%
	CD3E	0,88	0,66–1,17	0,38	100%
	GZMK	0,79	0,61–1,04	0,092	100%
	GZMB	0,82	0,61–1,11	0,2	100%
	IL2RG	1,14	0,86–1,53	0,36	100%
	TAGAP	1,21	0,92–1,59	0,16	100%
	LAG3	0,71	0,52–0,96	0,024	>50%
	NKG7	0,87	0,65–1,17	0,35	100%
	CIITA	0,87	0.66–1,14	0,31	100%
	CCL5	0.79	0,6–1,04	0,093	100%
	IDO1	0.83	0.63–1,11	0,21	100%
	CXCL10	0,82	0,61–1,1	0.19	100%
	HLA-DRA	1,11	0,82–1,52	0,49	100%
	STAT1	1,24	0,95–1,63	0,11	100%
	HLA-E	1,39	1,04–1,86	0,024	>50%
***IFN gamma*** ***genes***	CXCL9	0,73	0,55–0,58	0,034	>50%
	IFNG	0,71	0,54–0,93	0,013	>50%
	IDO1	0.83	0.63–1,11	0,21	100%
	CXCL10	0,82	0,61–1,1	0.19	100%
	IRF9	1,25	0,95–1,64	0,11	100%
	HLA-DRA	1,11	0,82–1,52	0,49	100%
	STAT1	1,24	0,95–1,63	0,11	100%
***CTL level***	GZMB	0,82	0,61–1,11	0,2	100%
	GZMA	0,84	0,63–1,12	0,23	100%
	PRF1	1,19	0,9–1,56	0,22	100%
	CD8A	0,83	0,62–1,1	0,19	100%
	CD8B	0,81	0,6–1,08	0,15	100%
***HLA genes***	HLA-B	1,26	0,96–1,65	0,095	100%
	HLA-A	1,15	0,85–1,57	0,37	100%

Table shows the list of individual genes belonging to the four previous published immune gene signatures. HR < 1 discriminates a risk reduction. The 95% confidence interval (CI) and p value are displayed. p value < 0.05 and FDR (False Discovery Rate) to correct for multiple hypothesis testing are displayed.

**Table 4:** Clinical outcome of individual immune genes from the four immune gene signatures in terms of overall survival (OS) of ESCC patients.

Signature	OS N= 81 (All stages)				
	Gene symbol	HR	CI	pValue	FDR
***Expanded*** ***immune*** ***genes***	CXCL13	1,68	0,57–4,92	0,34	100%
	CD2	2,5	1,08–5,74	0,027	>50%
	CD3D	2,56	1,06–6,18	0,03	>50%
	CXCR6	2,37	1,05–5,34	0,032	>50%
	CD3E	2,71	1,11–6,62	0,023	>50%
	GZMK	2,05	0,82–5,13	0,12	100%
	GZMB	2,34	1,03–5,33	0,038	>50%
	IL2RG	2,27	0,9–5,74	0,076	100%
	TAGAP	2,71	1,11–6,63	0,023	>50%
	LAG3	1.92	0,83–4,45	0,12	100%
	NKG7	2,41	0,99–5,87	0.046	>50%
	CIITA	1,46	0,62–3,44	0,38	100%
	CCL5	2,16	0,85–5,47	0,097	100%
	IDO1	2,1	0,91–4,85	0,076	100%
	CXCL10	2,18	0,8–5,89	0,12	100%
	HLA-DRA	2,12	0,87–5,2	0,093	100%
	STAT1	2,63	1,03–6,71	0,036	>50%
	HLA-E	3,53	1,46–8,51	0,0029	10%
***IFN gamma*** ***genes***	CXCL9	2,28	0,94–5,53	0,062	100%
	IFNG	2,78	1,04–7,43	0,034	>50%
	IDO1	2,1	0,91–4,85	0,076	100%
	CXCL10	2,18	0,8–5,89	0,12	100%
	IRF9	2,53	1,02–6,29	0,038	>50%
	HLA-DRA	2,12	0,87–5,2	0,093	100%
	STAT1	2,63	1,03–6,71	0,036	>50%
***CTL level***	GZMB	2,34	1,03–5,33	0,038	>50%
	GZMA	2,81	1,18–6,67	0,015	50%
	PRF1	2,75	1,14–6,64	0,02	>50%
	CD8A	1,72	0,77–3,84	0,18	100%
	CD8B	2,89	0,98–8,51	0,045	>50%
***HLA genes***	HLA-B	3,67	1,42–9,45	0,0041	20%
	HLA-A	2,52	1,1–5,75	0,024	>50%

Table shows the list of individual genes belonging to the four previous published immune gene signatures. HR < 1 discriminates a risk reduction. The 95% confidence interval (CI) and p value are displayed. p value < 0.05 and FDR (False Discovery Rate) to correct for multiple hypothesis testing are displayed.

**Figure 5:**
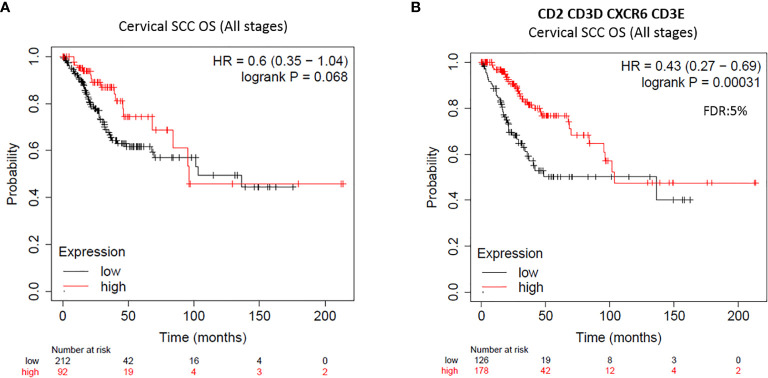
Expression of *CD2*, *CD3D*, *CD3E*, *CXCR6* immune gene combination shows better clinical outcome of CSSC patients. Clinical outcome of survival plots in CSCC at all stages (n = 254) with transcriptomic expression of all four previous published immune signatures **(A)** and with the new immune gene-set combination *CD2*, *CD3D*, *CD3E*, *CXCR6* (HR < 0.65 and FDR = 5%) **(B)**. HR for risk of death and OS are displayed. HR < 0.65 discriminates a risk reduction. FDR is also displayed.

In CSCC we found that the expression of the analyzed immune signatures (Expanded immune genes, IFN gamma genes, CTL genes, and HLA genes) were associated with favorable OS in all stages (HR=0.6; 95% CI=0.35–1.04; log rank p=0.068) (**Figure 5A** and **Table 5**), showing similar results with that in the of case of HNSCC. Most individual genes were associated with favorable outcome at all stages (n: 254; **Table 5**), and the previously selected genes showed the most favorable prediction capacity: CD2 (HR=0.41; 95% CI=0.26−0.66; log rank p= 0,0001), CD3D (HR=0.39; 95% CI=0.24–0.64; log rank p= 8,5e^−05^), CD3E (HR=0.42; 95% CI=0.26–0.67; log rank p=0.0002), and *CXCR6* (HR=0.44; 95% CI=0.27–0.70; log rank p=0.0004). Of note, in the CSCC cohort, we identified that expression of LAG3 was associated with very favorable outcome (HR=0.3; 95% CI=0.15−0.61; log rank p= 0,0004). We next evaluated the combined effect of the identified immune gene signature (combining *CD2*, *CD3D*, *CD3E*, and *CXCR6*), demonstrating a favorable prognosis (HR=0.43; 95% CI=0.27–0.69; log rank p=0.00031) with a low FDR (<5%) (**Figure 5B**).

**Table 5:** Clinical outcome of individual immune genes from the four immune gene signatures in terms of overall survival (OS) of CSCC patients.

Signature	OS N = 254 (All stages)				
	Gene symbol	HR	CI	pValue	FDR
***Expanded*** ***immune*** ***genes***	CXCL13	0,55	0,35–0,88	0,012	>50%
	**CD2**	**0,41**	**0,26–0,66**	**0,0001**	**2%**
	**CD3D**	**0,39**	**0,24–0,64**	**8,5e-05**	**1%**
	**CXCR6**	**0,44**	**0,27–0,70**	**0,0004**	**5%**
	**CD3E**	**0,42**	**0,26–0,67**	**0.0002**	**2%**
	GZMK	0,47	0,27–0,81	0,0059	>50%
	GZMB	0,41	0,22–0,78	0,005	>50%
	IL2RG	0,62	0,39–1,00	0,046	>50%
	TAGAP	0,64	0,39–1,06	0.079	100%
	LAG3	0,3	0,15–0,61	0,0004	5%
	NKG7	0,45	0,24–0,84	0,01	>50%
	CIITA	0,54	0,33–0,89	0,014	>50%
	CCL5	0,47	0,29–0,76	0,0015	20%
	IDO1	0,5	0,28–0,89	0,017	>50%
	CXCL10	0,63	0,39–1,01	0,053	100%
	HLA-DRA	0,48	0,3–0,79	0,0033	50%
	STAT1	0,65	0,4–1,03	0,065	100%
	HLA-E	0,68	0,41–1,12	0,13	100%
***IFN gamma*** ***genes***	CXCL9	0,42	0,22–0,8	0,0069	>50%
	IFNG	0,36	0,19–0,71	0,002	20%
	IDO1	0,5	0,28–0,89	0,017	>50%
	CXCL10	0,63	0,39–1,01	0,053	100%
	IRF9	0,63	0,39–1,00	0,049	>50%
	HLA-DRA	0,48	0,3–0,79	0,0033	50%
	STAT1	0,65	0,4–1,03	0,065	100%
***CTL level***	GZMB	0,41	0,22–0,78	0,005	>50%
	GZMA	0,48	0,25–0,92	0,023	>50%
	PRF1	0,56	0,34–0,94	0,026	>50%
	CD8A	0,45	0,28–0,72	0,0006	10%
	CD8B	0,49	0,3–0,78	0,002	20%
***HLA genes***	HLA-B	0,7	0,42–1,15	0,15	100%
	HLA-A	0,66	0,41–1,07	0,091	100%

Table shows the list of individual genes belonging to the four previous published immune gene signatures. HR < 1 discriminates a risk reduction. The 95% confidence interval (CI) and p value are displayed. p value < 0.05 and FDR (False Discovery Rate) to correct for multiple hypothesis testing are displayed.

The bold values represent those genes that stand out with the best clinical outcome (HR < 0.65, FDR < 10%, p < 0.05).

The assessment of the new immune gene signature (*CD2*, *CD3D*, *CD3E*, and *CXCRC6*) in the CSCC cohort per tumor stage was not possible because sample size was too low for a meaningful analysis when we filtered data by stage.

### Expression of *CD2*, *CD3D*, *CD3E*, and *CXCR6* Is Linked to a High Expression of Memory CD8+ T Cells, Activated NK Cells, and M1 Macrophages in CSCC

**Figure 6:**
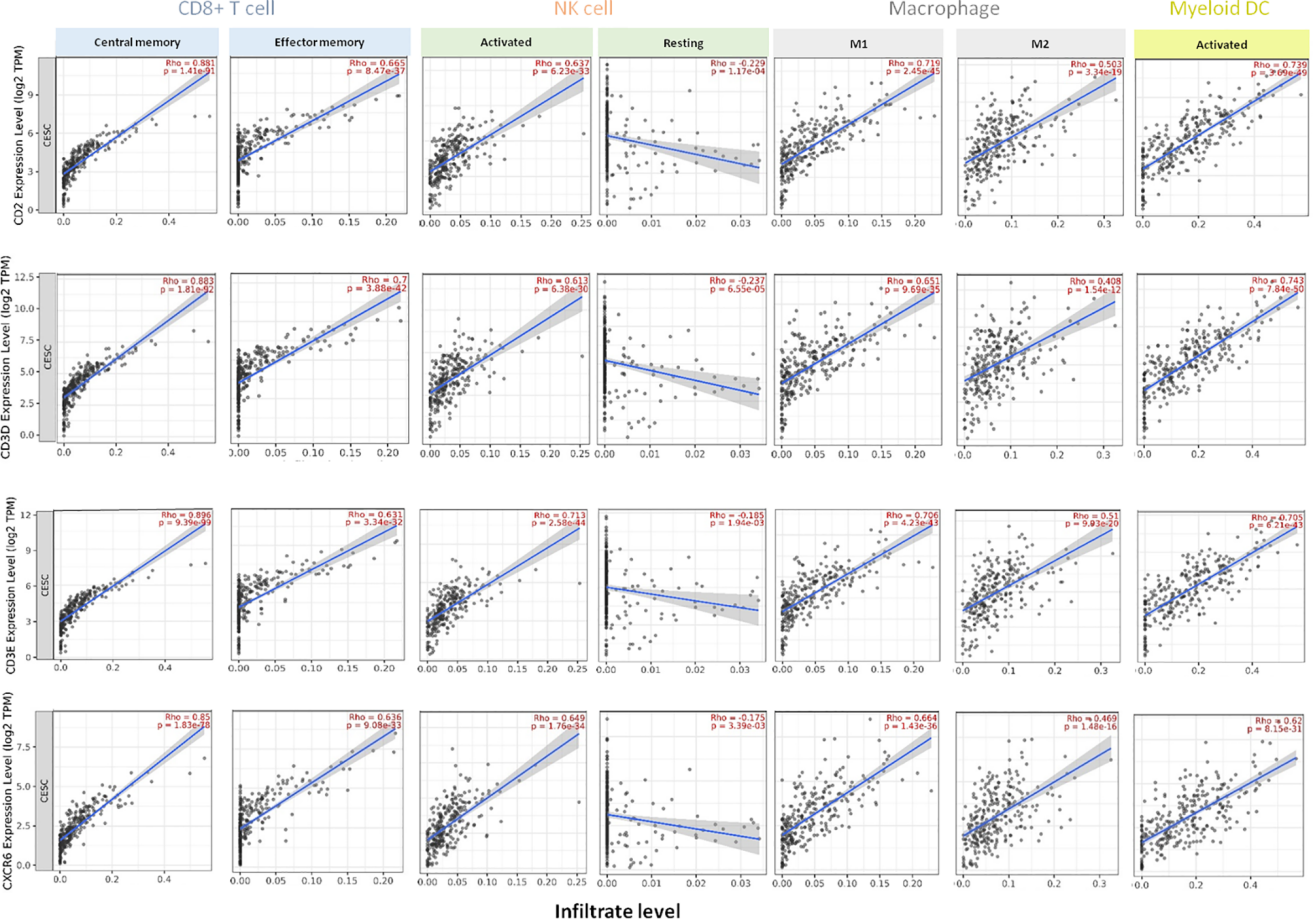
Association of *CD2*, *CD3D*, *CD3E*, *CXCR6* transcriptomic expression with specific tumor-infiltrating immune cell populations in CSCC patients. Expression of each gene in association with the presence of CD8+ T cells (central memory and effector memory subsets), NK cells (activated or resting), macrophages (M1 and M2 types), and activated myeloid dendritic infiltrates in CSSC patients (n = 254). Each panel presents the relationship between infiltrates estimation value with the purity-adjusted spearman’s correlation parameter Rho and gene expression, join p value < 0.001. Rho value > 0 represents a positive correlation, and Rho < 0 represents a negative correlation. Association with immune cell populations was provided by *TIMER 2.0* software and was correlated with transcriptome expression level of each immune gene.

Since *CD2*, *CD3D*, *CD3E*, and *CXCR6* predict very favorable prognosis in CSCC, we evaluated the association of this new signature at transcriptomic level with the presence of tumor-infiltrating immune cell populations in CSCC. All genes showed a negative correlation with tumor purity. As in the case of HNSCC patients, we found a positive correlation between the gene expression of *CD2*, *CD3D*, *CD3E*, and *CXCR6* and CD8^+^ T cells (central and effector memory subpopulations), and the strongest correlation was observed for the central memory CD8^+^ T cells (*CD2:* Rho=0.881, *CD3D:* Rho=0.883, *CD3E:* Rho=0.896, and *CXCR6:* Rho=0.850) (**Figure 6**). A positive association was also observed for the CD8+ effector memory subpopulation (*CD2:* Rho=0.665, *CD3D:* Rho=0.700, *CD3E:* Rho=0.631, and *CXCR6:* Rho=0.636) (**Figure 6**).

In case of the NK cell population, the strongest correlation was found with the activated fraction (*CD2:* Rho=0.637, *CD3D:* Rho=0.613, *CD3E:* Rho=0.713, and *CXCR6:* Rho=0.649). In the macrophage population, the highest association was identified for the M1 group (*CD2:* Rho=0.719, *CD3D:* Rho=0.651, *CD3E:* Rho=0.706, and *CXCR6:* Rho=0.664) compared with the M2 macrophage population (*CD2:* Rho=0.503, *CD3D:* Rho=0.408, *CD3E:* Rho=0.510, and *CXCR6:* Rho=0.469). For activated dendritic cells, the results were as follow: *CD2:* Rho=0.739, *CD3D:* Rho=0.743, *CD3E:* Rho=0.705, and *CXCR6:* Rho=0.620) (**Figure 6**).

### Clinical Outcome in HNSCC and CSSC With Expression of *CD2, CD3D, CD3E, CXCR6* and High Mutation Burden

**Figure 7:**
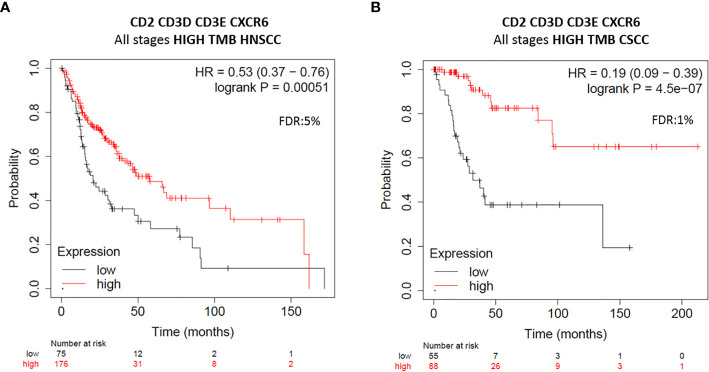
Clinical outcome analysis of *CD2*, *CD3D*, *CD3E*, *CXCR6* immune gene signature expression in HNSCC and CSCC tumors with high mutational burden. Clinical outcomes of survival plots of new immune gene signature with improved favorable prognosis at all stages of HNSCC tumors (n = 251) **(A)** and CSCC tumors (n = 143) **(B)** with high mutational burden (TMB) are displayed. Red line represents survival of patients whose tumors harbor high gene expression levels, and those with low gene expression levels show in black line. Number of patients at risk at every time (months), with high (in red) and low gene expression (in black), is displayed. HR for risk of death and OS are displayed. HR < 0.65 discriminates a risk reduction. FDR is also displayed. The gene combination is displayed at the top of each figure.

As the mutational burden has been associated with response to immune-modulatory drugs (18, 19), we explored if the expression of the identified immune signature *CD2*, *CD3D*, *CD3E*, *CXCR6* was able to predict a better outcome in HNSCC and in CSCC with high mutational burden. In HNSCC with high mutational burden, the presence of the immune signature was able to predict favorable OS (n=251; HR=0.53; 95% CI=0.37–0.76; log rank p=0.00051; FDR=5%) (**Figure 7A**). Similarly, in CSCC tumors with high mutational burden, the presence of the immune signature predicted strongly favorable survival (n=143; HR=0.19; 95% CI=0.09–0.39; log rank p=4.5e-07, FDR=1%) (**Figure 7B**). This result suggests that outcome prediction of the aforementioned immune signature is much more effective in HSCC and CSCC tumors with high mutational burden.

## Discussion

In the present article we describe immune genomic signatures associated with favorable outcome in HNSCC and CSCC. Identification of genomic immune correlates that predict outcome as an indirect measure of immune activation is a key task in oncology.

SCCs comprise a large family of tumors from epithelial tissues that arise from different locations but that share some common biological characteristics like genomic instability, dysfunction of DNA repair mechanisms, or relative sensibility to therapeutic agents that induce DNA damage or affect DNA repair mechanisms (1). In addition, some of them have shown to be more sensitive to immunologic agents probably due to the retained viral antigens produced by the presence of HPV infection (4, 5).

Using previously described transcriptomic signatures associated with immune activation, we aimed to identify genes that were linked with favorable outcome in HNSCC patients. We found a correlation with outcome of most of the signatures except for the HLA one. The fact that some genes correlated better than the whole signature let us explore a combination of transcripts that could increase the prediction capacity, and this was the case for a signature that included only four genes: *CD2*, *CD3D*, *CD3E*, and *CXCR6*. Of note, these findings were reproduced in CSCC and with minor significance in esophagus tumors. The association between gene expression profile and clinical outcome in patients selecting by HPV condition was not available with KM Plotter Online Tool in the case of the HNSCC cohort of patients.

A remarkable finding is the fact that the identified results were not reproduced in other squamous cell lung cancers. These results, although surprising, confirm the heterogeneity of tumors at a location and histology level (28, 29). Probably, the results have been conditioned by the presence of HPV in these two indications: HNSCC and CSCC (most CSCC tumors are HPV positive), a situation that is not observed in other squamous cell tumors like esophagus or lung. These tumors lack the presence of HPV infection, so they do not exhibit the viral neoantigens or molecules likely recognized by the identified immune cell populations in HNSCC and CSCC.

The immune gene signature comprised several genes. CD3D and CD3E are part of the TCR-CD3 complex present on T-lymphocyte cell surface (30, 31). CD3 chains contain immunoreceptor tyrosine-based activation motifs (ITAMs) in their cytoplasmic domain, and after T-cell receptor engagement, they transmit the activation signaling by phosphorylation of SRC (30). In this context, the presence of these two genes is an indirect measure of the existence of activated T cells. It is not surprising to see that their presence has also been described as linked with prognosis (32). CD2 is a cell adhesion protein expressed on the T cell and NK cell surface and has been used as a specific marker for these two populations (33). Finally, CXCR6 has been described as a chemokine associated with the activation of IFN gamma effector cells, therefore can constitute an adequate marker to select active T cells (34).

The presence of the selected genes correlated with low tumor purity in HNSCC and CSCC. Moreover, we observed a very clear and strong association with the presence of populations of cells involved in adaptive immune cell response including activated T cells and dendritic cells. In addition, we observed an increase in M1 macrophages and in activated NK cells, demonstrating that the innate immunity was also present and therefore can have a role in the antitumoral action. This finding is relevant as a single signature of four genes can identify an immunologic state that is linked with a favorable prognosis compromising an adaptive and innate activated immune response. Unfortunately, no evaluation of immune populations in esophageal SCC was performed, since data are not publicly available.

When exploring differences between HNSCC HPV-positive and HPV-negative tumors, we did not observe major discrepancies beyond the fact that HPV-positive tumors had a stronger presence of the described immune cells (35). In fact, almost all CSCC tumors are HPV positive. As mentioned before, tumors in which a viral infection is a key oncogenic event can have a wider presence of neoantigens and therefore an increase presence of immune activated cells, what suggests that could be more sensitive to agents that modulate the immune system. In fact, we observed that the expression of the new immune signature correlated with the same composition of tumoral immune cell infiltrates in HNSCC and CSCC (see and compare **Figure 3** and **Figure 6**), maybe since both types of tumors shared the same pattern of neoantigens.

A relevant finding of our study is the discrimination in outcome between those tumors with a high tumor mutational burden (TMB). In this context, not all tumors with TMB respond to immune modulators, which suggests that the identification of biomarkers within this population will also be of help.

We acknowledge that our study has limitations. This is an *in silico* analysis using datasets from different sources. However, all the datasets included in this study are publicly available and have been incorporated in several studies that support their consideration as representative of the general population. Of note, the compilation and integration of molecular biology and clinical data into the available datasets is sometimes scarce. For instance, we could not explore if in terms of expression our new signature had different prediction in the HNSCC cohort per HPV status. This was not the case in CSCC where all patients are HPV positive.

In conclusion, we describe a set of genes that are able to identify immune activated tumors involving adaptive and innate immune response, associated with favorable prognosis in HNSCC and CSCC. Prospective studies should be performed to confirm these results.

## Data Availability Statement

Publicly available datasets were analyzed in this study. This data can be found here: https://kmplot.com/analysis/, http://cistrome.org/TIMER/download.html.

## Author Contributions

AO, CS-L, MB-P, and AM have contributed to conception and design of the study. AO has performed the main design of the work. CS-L and MB-P have carried out the acquisition, analysis, or interpretation of data for the work. All authors contributed to the article and approved the submitted version.

## Funding

This work has been supported by Instituto de Salud Carlos III (PI16/01121 and PI19/00808), ACEPAIN; Diputación de Albacete, CIBERONC and CRIS Cancer Foundation (to AO). Ministry of Economy and Competitiveness of Spain (BFU2015-71371-R), the Instituto de Salud Carlos III through the Spanish Cancer Centers Network Program (RD12/0036/0003) and CIBERONC, the scientific foundation of the AECC and the CRIS Foundation (to AP). The work carried out in our laboratories receive support from the European Community through the regional development funding program (FEDER).

## Conflict of Interest

The authors declare that the research was conducted in the absence of any commercial or financial relationships that could be construed as a potential conflict of interest.

## Publisher’s Note

All claims expressed in this article are solely those of the authors and do not necessarily represent those of their affiliated organizations, or those of the publisher, the editors and the reviewers. Any product that may be evaluated in this article, or claim that may be made by its manufacturer, is not guaranteed or endorsed by the publisher.

## Supplementary Material

Supplementary Figure 1 Clinical outcome analysis of *CD2*, *CD3D*, *CD3E*, *CXCR6* immune gene signature expression in lung SCC and esophageal SCC tumors. Clinical outcomes of survival plots of new immune gene signature with no prediction at all stages of lung SCC tumors (n = 501) **(A)** and poor prognosis at all stages in esophageal SCC tumors (n = 81) **(B)** are displayed. Red line represents survival of patients whose tumors harbor high gene expression levels, and black line represents those with low gene expression levels. Number of patients at risk at every time (months) is displayed. HR for risk of death and overall survival (OS) are displayed. HR < 0.65 discriminates a risk reduction. FDR is also displayed. The gene combination is displayed at the top of each figure.
