# Supplementary figures and images for: Transcriptomic Correlates of Immunologic Activation in Head and Neck and Cervical Cancer
Source: Front Oncol. 2021 Oct 6;11:714550. doi: 10.3389/fonc.2021.714550 (PMC8527851; doi:10.3389/fonc.2021.714550)

Supplementary Figure 1


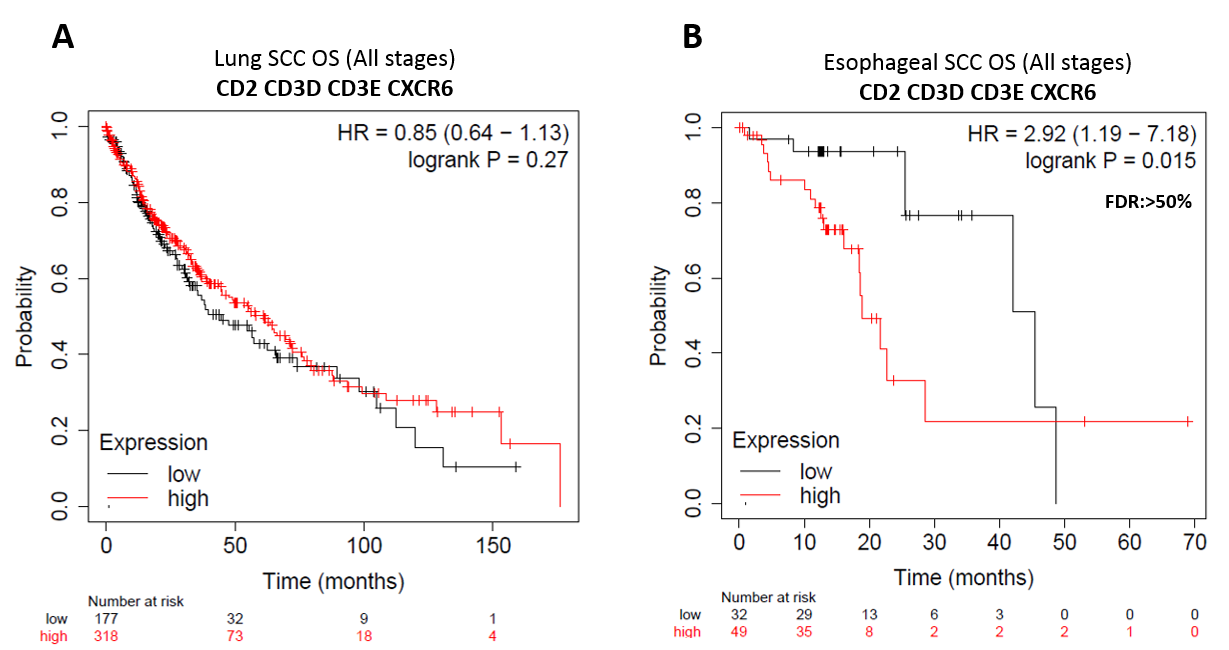

Supplement: Supplementary Figure 1 — Clinical outcome analysis of CD2, CD3D, CD3E, CXCR6 immune gene signature expression in lung SCC and esophageal SCC tumors. Clinical outcomes of survival plots of new immune gene signature with no prediction at all stages of lung SCC tumors (n = 501) (A) and poor prognosis at all stages in esophageal SCC tumors (n = 81) (B) are displayed. Red line represents survival of patients whose tumors harbor high gene expression levels, and black line represents those with low gene expression levels. Number of patients at risk at every time (months) is displayed. HR for risk of death and overall survival (OS) are displayed. HR < 0.65 discriminates a risk reduction. FDR is also displayed. The gene combination is displayed at the top of each figure. [file DataSheet_1.docx]
